# British Home and Hospital for Incurables, Streatham

**Published:** 1902-05-24

**Authors:** 


### BRITISH HOME AND HOSPITAL FOR INCURABLES, STREATHAM.

### CORONATION GIFT TO H.M. QUEEN ALEXANDRA.

This home being the first charitable institution to receive the support and patronage of Queen Alexandra, it is only natural that the Board of Management should take advantage of the favourable opportunity afforded them by the Coronation to lay their claims for support before the public. They have accordingly, with Her Majesty's sanction and approval, decided to raise a fund to establish pensions commemorative of her coronation. The names of all subscribers will be placed in an album which Her Majesty has graciously promised to accept on her proposed visit to the home in the course of the summer. Subscriptions amounting to £2,200 have already been received, and it is hoped that a total of £5,000 will be reached before the fund is closed.

Annual General Meeting.

The subscribers to the home having all received information concerning the fund for the Coronation gift, more than ordinary interest attached to the annual general meeting, held on May 13th, but the attendance was scarcely worthy of the occasion.

The Chairman, Mr. F. A. Bevan, moved the adoption of the report, and said that it was in every way an entirely satisfactory one. Looked at from the treasurer's point of view it was especially so, the receipts for the year amounting to over £26,733, which was the largest total they had ever attained to in any previous year. He wished, however, to lay emphasis upon the fact that this satisfactory result was almost entirely owing to two large legacies which they had received of £5,000 each, and he trusted that the subscribers would look upon this additional £10,000 as an entirely exceptional occurrence and by no means consider that the home was now so rich that they might withdraw or curtail their subscriptions. Owing to the extra funds they had received, combined with their celebration of the Coronation, the chairman stated that they had decided to elect some extra pensioners, and instead of ten they had elected fourteen.

The Chairman made a sympathetic allusion to the loss the board had sustained during the past year in the death of Lieutenant-General Sir Francis Norman, K.C.B., and mentioned that General Sir Alexander Montgomery-Moore had been elected to fill the vacancy.

Referring to the proposed Coronation gift to the Queen, the Chairman drew attention to the fact that the home was one of the two institutions to which Her Majesty had devoted the proceeds of the sale of Canon Fleming's sermon preached on the occasion of the Duke of Clarence's death, and that the home had benefited to the amount of over £800 by their portion of this sale. Whilst heartily applauding the Coronation gift to the King, for which the Lord Mayor and his fellow Mayors were making so stirring an appeal, the chairman pointed out that the British Home for Incurables would derive no benefit whatever from such a gift, and therefore, although he earnestly hoped that the appeal of the Lord

Mayor would meet with a liberal response, he also hoped that their own appeal would likewise receive a liberal answer. It was difficult to realise, he went on, the enormous difference that the yearly pension of £20 made in the lives of the poor incurables who were almost entirely helpless; often it meant the difference between a miserable existence and one of comparative ease and comfort.

The Chairman ended by drawing an interesting comparison between the home as they knew it now, containing ample accommodation for 80 patients and situated in an ideal position in the highest part of Streatham, and the home as it was when he first became treasurer, when it consisted of two or three leasehold houses in Clapham.

The usual business routine having been quickly dispatched, the meeting adjourned for tea, which was awaiting them at the other end of the large hall.

